# Rapid Antisuicidal Effects of Intravenous Ketamine as an Emergency Intervention in Outpatient and Consultation-Liaison Psychiatry Settings

**DOI:** 10.1192/j.eurpsy.2026.10940

**Published:** 2026-07-31

**Authors:** P. Argitis, M. Demetriou, V. Anagnostopoulou, F. Mourka, Z. Chaviaras

**Affiliations:** Psychiatric, General Hospital of Corfu, Corfu, Greece

## Abstract

**Introduction:**

Acute suicidal ideation after a suicide attempt is a psychiatric emergency requiring immediate treatment. IV ketamine (0.5 mg/kg), with rapid onset, may offer such benefit. This study examines its efficacy and safety as a first-line intervention for swiftly resolving suicidal ideation in patients post-attempt, including in outpatient and consultation-liaison psychiatry settings.

**Objectives:**

The primary aim was to assess the ultra-rapid (within 24h) antisuicidal effects of a single IV ketamine infusion (0.5 mg/kg) in patients with active suicidal ideation post-attempt. Secondary aims included evaluating safety, tolerability, and the practical advantages of IV ketamine over intranasal esketamine, given cost and accessibility constraints in the Greek healthcare system.

**Methods:**

A retrospective chart review was conducted on 42 adult patients (mean age 36.8 ± 9.2 years; 62% female) who, following a recent suicide attempt and presenting with active suicidal ideation, **immediately** received a single IV ketamine infusion (0.5 mg/kg over 40 minutes) as an **emergency intervention** in an outpatient or consultation-liaison setting. Suicidal ideation was assessed using item 10 (Suicidal thoughts, 0-6 scale) of the Montgomery-Åsberg Depression Rating Scale (MADRS) at baseline (pre-infusion), **critically within 24 hours post-infusion **, and at 7 days. Demographics, primary psychiatric diagnosis, and adverse events were also recorded.

**Results:**

A statistically significant and clinically profound reduction in mean MADRS item 10 scores was observed as early as 24 hours post-infusion (from M=4.5, SD=1.1 at baseline, to M=1.2, SD=0.9; mean reduction=3.3, 95% CI [2.9, 3.7], p<0.001), underscoring the **immediate therapeutic relief**. This improvement was largely sustained at 7 days (M=1.5, SD=1.0, p<0.001 vs. baseline). A substantial 76.2% (32/42) of patients demonstrated a clinically significant response (≥50% reduction in MADRS item 10 or score ≤2) within the first 24 hours. Notably, 61.9% (26/42) achieved near-complete remission of suicidal ideation (MADRS item 10 score of 0 or 1) within this timeframe, compared to 0% at baseline. Adverse events were mild and transient (mild dissociative symptoms 28%, transient blood pressure elevation 11%, nausea 9%), with none leading to treatment discontinuation.

**Conclusions:**

A single IV ketamine infusion (0.5 mg/kg) given post-attempt provides ultra-rapid relief from suicidal ideation, proving safe and well-tolerated in outpatient and liaison psychiatry settings. Its lower cost and greater availability compared to esketamine in Greece strengthen its role as a vital, time-sensitive intervention for acute suicidal crises. The comparative advantages of IV ketamine regarding immediate availability and lower cost compared to esketamine in Greece further enhance its utility as a vital, deployable tool for managing acute suicidal crises.

**Disclosure of Interest:**

None Declared

